# Design of an FMCW radar baseband signal processing system for automotive application

**DOI:** 10.1186/s40064-015-1583-5

**Published:** 2016-01-18

**Authors:** Jau-Jr Lin, Yuan-Ping Li, Wei-Chiang Hsu, Ta-Sung Lee

**Affiliations:** Department of Electrical Engineering, National Changhua University of Education, 500 Changhua City, Taiwan; Department of Electrical and Computer Engineering, National Chiao Tung University, 300 Hsinchu City, Taiwan

**Keywords:** Automotive radar, Frequency modulated continuous wave (FMCW), Multi-target detection, Radar waveform, Antenna array

## Abstract

For a typical FMCW automotive radar system, a new design of baseband signal processing architecture and algorithms is proposed to overcome the ghost targets and overlapping problems in the multi-target detection scenario. To satisfy the short measurement time constraint without increasing the RF front-end loading, a three-segment waveform with different slopes is utilized. By introducing a new pairing mechanism and a spatial filter design algorithm, the proposed detection architecture not only provides high accuracy and reliability, but also requires low pairing time and computational loading. This proposed baseband signal processing architecture and algorithms balance the performance and complexity, and are suitable to be implemented in a real automotive radar system. Field measurement results demonstrate that the proposed automotive radar signal processing system can perform well in a realistic application scenario.

## Background

Recently, the automotive radar systems have been employed in various active safety applications, such as adaptive cruise control, crash mitigation, and pre-crash sensing (Wenger 2005; Lachner 2009). The frequency modulated continuous wave (FMCW) technique (Barriok 1973; Stove 1992; Komarov and Smolskiy 2003) which is known for high resolution measurements has been widely used in the area of automotive radars or instrumentation and measurement (Woods et al. 1993; Stolle and Schiek 1997; Journet and Bazin 2000; Sahu and Gupta 2008). In (Woods et al. 1993), a microwave ranging system using a composite FMCW measurement technique was proposed. Also, adaptive spatial digital filtering was applied to the FMCW radar measurements to reduce the influence of clutter. A microwave FMCW radar for precision ranging of multiple targets was built in (Stolle and Schiek 1997) for real-time applications. The evaluation of the FMCW raw data is mainly based on a fast Fourier transform (FFT) and the phase information is extracted from the FMCW data. Thus, the method can enhance measurement accuracy with small phase errors. In (Journet and Bazin 2000), the FMCW technique applied to a prototype of a low-cost laser range finder was presented. The method for the measurement of distance and medium velocity using ultrasound based on the principle of FMCW technique was proposed in (Sahu and Gupta 2008). A more comprehensive wideband range-Doppler algorithm was proposed in (Wagner et al. 2013) using a new waveform design and a 2D-FFT to estimate the range and velocity information. The principle presented in (Wagner et al. 2013) allows the use of very broadband sweeps. The advantages of FMCW radars in comparison to pulse radars are the low measurement time and low peak-to-average power ratio. In automotive safety applications, the range and velocity information of individual targets requires being measured simultaneously and being updated in short time. By taking advantage of the emerging technologies, FMCW radars become feasible to realize signals generated and processed in real time for high performance automotive safety systems (Folster et al. 2005; Li et al. 2010).

The basic idea of FMCW automotive radars is to attain the range and velocity information from the beat frequency, which is composed of propagation delay and Doppler frequency (Winkler 2007; Rohling and Moller 2008). However, in multi-target detection, typical FMCW radars will suffer from the range-velocity ambiguity problem (Rohling and Meinecke 2001), which causes ghost targets and missed targets. Some FMCW waveforms were designed to solve this problem (Meinecke and Rohling 2000; Rohling and Meinecke 2001; Miyahara 2004; Rohling and Moller 2008; Bi and Du 2010). The methods in (Meinecke and Rohling 2000; Rohling and Meinecke 2001; Rohling and Moller 2008; Bi and Du 2010) decouple the range-velocity information by combining FMCW with another frequency modulation technique to resolve the ambiguity. These methods could maintain both the accuracy and the short measurement and processing time, but could potentially increase the RF front-end loading by generating these special waveforms. The multiple segments waveform (Miyahara 2004) is adopted to determine a unique pair of up and down beat frequencies for each real target by extending observation time, which could violate the short measurement and processing time requirement in high performance automotive radar systems. In this paper, a three-segment waveform with different slopes is proposed. This three-segment waveform design not only solves the range-velocity ambiguity problem, but also satisfies the short measurement and processing time constraint without increasing the RF front-end loading.

In addition to the range and velocity information in time and frequency domain, the azimuth angle information from spatial domain plays an important role in the automotive radar systems as well. Various angle estimation methods (Moon et al. 2003; Choi et al. 2011) are available to deal with angle estimation problems. In automotive radar systems, only a small number of snapshots are available for angle estimation in high mobility scenarios. The eigen-based angle estimator can separate targets with a similar beat frequency in a single snapshot (Häcker and Yang 2010). This method meets the requirement but needs a high computational effort, and generates a lot of wrong angle information. A low complexity maximum-likelihood angle estimator based on the phase-comparison technique (Huang et al. 2013) reduces the computational loading and is adopted in our system. However, due to overlapping between beat frequencies of different targets, the estimated angle information will be unreliable in multi-target detection. In this paper, the spatial filter design algorithm is applied to identify the overlapping targets. A minimum-norm method (Rao and Hari 1989) then distinguishes the overlapping targets. The minimum-norm method with a spatial filter algorithm has also been widely used in image processing areas (Uusitalo and Ilmoniemi 1997; Blankertz et al. 2007). The proposed detection architecture requires low pairing time and provides high reliability.

This paper is organized into sections as follow. In “FMCW waveform design”, the details of the three-segment waveform design and the concept of the pairing mechanism are present. In “Multi-target detection architecture and algorithms”, the proposed baseband signal processing architecture and algorithms, which include the paring mechanism and the multi-target detection algorithms, will be discussed. In “Simulation and experiment results”, the high performance of the proposed baseband architecture and algorithms will be demonstrated and discussed. In particular, field measurement results demonstrate the efficacy of the proposed baseband signal processing architecture and algorithms. Finally, we will conclude this paper in “Conclusions”.

## FMCW waveform design

**Fig. 1:**
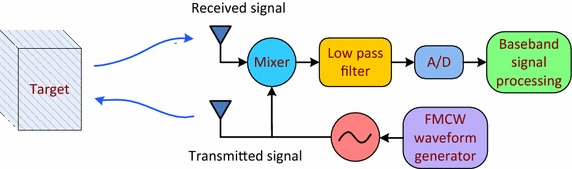
Block diagram of an FMCW system

An FMCW system is shown in Fig. 1 which consists of a transmitter, a receiver, a mixer and an analog-to-digital converter (A/D). A modulated signal is transmitted and received through antennas, and the transmitted and received signals are multiplied in the time domain and processed.

### Signal model

According to (Barriok 1973; Stove 1992; Komarov and Smolskiy 2003; Winkler 2007) the transmitted signal of an FMCW radar system can be modeled as

1 $$s_{T} (t) = A_{T} \cos \left( {2{{\pi }}f_{c} t + 2{{\pi }}\int_{0}^{t} {f_{T} (\tau )d\tau } } \right),$$

where $f_{T} (\tau ) = \tfrac{B}{T} \cdot \tau$ is the transmit frequency as a linear function of time, *f*_*c*_ is the carrier frequency, *B* is the bandwidth, *A*_*T*_ represents the transmitted signal amplitude, and *T* is the time duration. Considering a reflected signal with a time delay $t_{d} = 2 \cdot \tfrac{{R_{0} + vt}}{c}$ and Doppler shift $f_{D} = - 2 \cdot \tfrac{{f_{c} v}}{c}$, the receive frequency can be expressed as

2 $$f_{R} (t) = \frac{B}{T}\left( {t - t_{d} } \right) + f_{D} ,$$

where *R*_0_ is the range at $t = 0$, *v* is the target velocity, and *c* is the speed of light. The received signal can be described as

3 $$\begin{aligned} S_{R} (t) &= A_{R} \cos \left( {2{{\pi }}f_{c} \left( {t - t_{d} } \right) + 2{{\pi }}\int_{0}^{t} {f_{R} (\tau )d\tau } } \right) \hfill \\ & = A_{R} \cos \left\{ {2{{\pi }}\left( {f_{c} \left( {t - t_{d} } \right) + \tfrac{B}{T}\left( {\tfrac{1}{2}t^{2} - t_{d} \cdot t} \right) + f_{D} \cdot t} \right)} \right\}. \hfill \\ \end{aligned}$$

Here, *A*_*R*_ represents the received signal amplitude, which is dependent on antenna gains, transmitted power, and the target’s distance and radar cross section (RCS). To obtain information of the Doppler frequency and beat frequency, *S*_*T*_(*t*) and *S*_*R*_(*t*) are mixed by multiplication in the time domain, and passed to a low-pass filter (LPF). The intermediate frequency (IF) signal *S*_*IF*_(*t*) of the LPF output is then obtained for the up ramp as

4 $$ S_{IF} (t) = \frac{1}{2}\cos \left( {2{{\pi }}\left( {f_{c} \cdot \frac{{2R_{0} }}{c}} \right) + 2{{\pi }}\left( {\frac{{2R_{0} }}{c} \cdot \frac{B}{T} + \frac{{2f_{c} v}}{c}} \right)t} \right). $$

Similarly, the IF signal *S*_*IF*_(*t*) of the LPF output can be obtained for the down ramp as follows

5 $$S_{IF} (t) = \frac{1}{2}\cos \left( {2{{\pi }}\left( {f_{c} \cdot \frac{{2R_{0} }}{c}} \right) + 2{{\pi }}\left( { - \frac{{2R_{0} }}{c} \cdot \frac{B}{T} + \frac{{2f_{c} v}}{c}} \right)t} \right).$$

Hence, two time-dependent frequency terms called beat frequency appear in the spectrum of the baseband signal

6 $$\left\{ {\begin{array}{l} {f_{bu} = \frac{{2R_{0} }}{c} \cdot \frac{B}{T} + \frac{{2f_{c} v}}{c}} \\ {f_{bd} = - \frac{{2R_{0} }}{c} \cdot \frac{B}{T} + \frac{{2f_{c} v}}{c}} \\ \end{array} } \right..$$

**Fig. 2:**
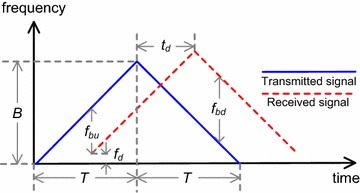
The received and transmitted frequencies of a triangular waveform for the FMCW radar system

We can then use these frequencies to solve for *v* and *R*_0_. Figure 2 shows the receive and transmit frequencies of the triangular waveform for the FMCW radar system, where *f*_*bu*_ and *f*_*bd*_ denote the up ramp beat frequency and down ramp beat frequency, respectively.

### Waveform design

**Fig. 3:**
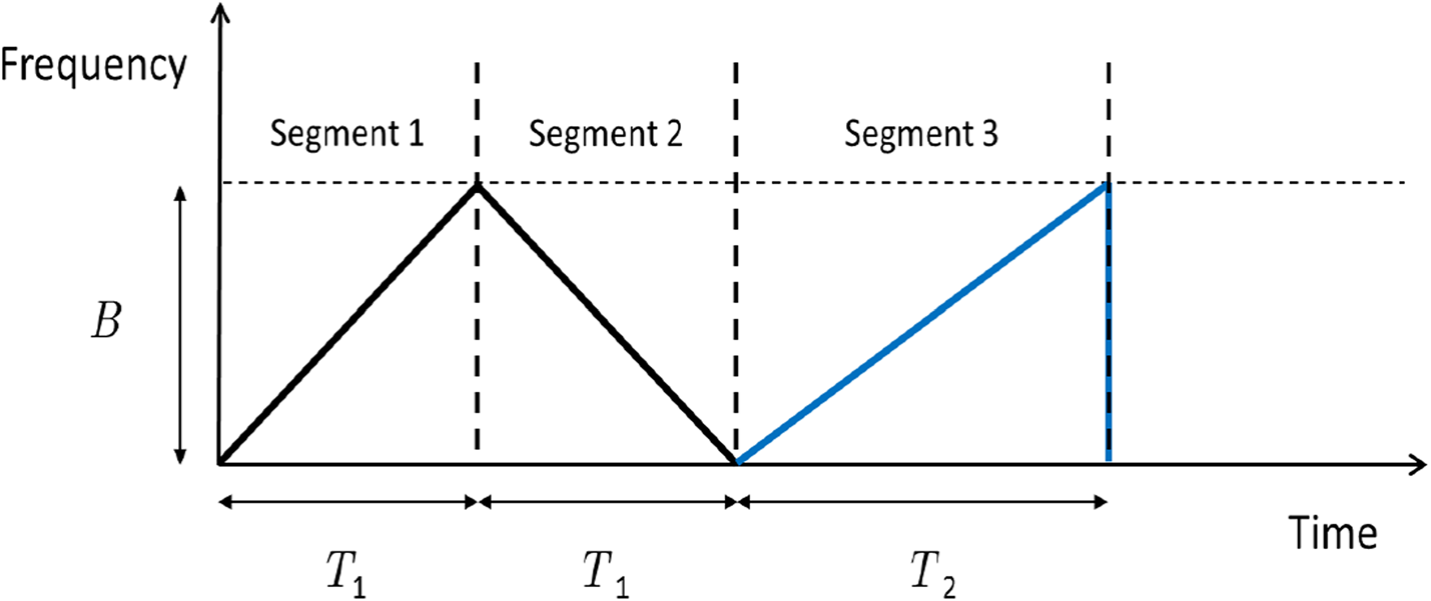
The proposed three-segment waveform

To satisfy the accuracy and the short measurement time without increasing the RF front-end loading, the three-segment waveform with different slopes shown in Fig. 3 is adopted, where the chirp frequency bandwidth is *B*, (a) the Segment 1 is the up ramp with ramp time *T*_1_, (b) the Segment 2 is the down ramp with ramp time *T*_1_, and (c) the Segment 3 is the check ramp with ramp time *T*_2_. The third ramp with different B/T ratio is intended to provide another aspect to verify if the up and down beat frequencies are paired correctly. First, the IF signal *S*_*IF*_(*t*) of different segments is transformed into the frequency domain by taking a 1-D FFT (Rohling and Moller 2008). Then, the data will go through the constant false alarm rate (CFAR) process (Weiss 1982; Rohling 1983; Touzi et al. 1988) to suppress the ghost targets. The order statistics (OS) CFAR (Rohling 1983) is adopted in our system, which provides good immunity to interfering targets. After the CFAR procedure, each measured beat frequency contains information about target range and velocity in an ambiguous way. Therefore, by pairing the beat frequency of different ramps, the range and velocity information of each individual target can be derived. The *k*th beat frequencies of different segments (Rohling and Moller 2008) can be expressed as

7 $$f_{bu,k} = \frac{{2R_{0,k} }}{c} \cdot \frac{B}{{T_{1} }} + \frac{{2f_{c} v_{k} }}{c},$$

8 $$f_{bd,k} = - \frac{{2R_{0,k} }}{c} \cdot \frac{B}{{T_{1} }} + \frac{{2f_{c} v_{k} }}{c},$$

9 $$f_{bc,k} = \frac{{2R_{0,k} }}{c} \cdot \frac{B}{{T_{2} }} + \frac{{2f_{c} v_{k} }}{c},$$

where *c* is the speed of light, *f*_*c*_ is the carrier frequency, *R*_0,*k*_ is the range information, *v*_*k*_ is the velocity information, *f*_*bu*,*k*_ is the *k*th up ramp beat frequency, *f*_*bd*,*k*_ is the *k*th down ramp beat frequency, and *f*_*bc*,*k*_ is the *k*th check ramp beat frequency. By pairing the beat frequencies of the up and down ramps, tentative estimates of the range and velocity information can be respectively expressed as

10 $$R_{0,te,ij} = \frac{{cT_{1} (f_{bu,i} - f_{bd,j} )}}{4B},$$

11 $$v_{te,ij} = \frac{{c(f_{bd,i} + f_{bu,j} )}}{{4f_{c} }},$$

where *R*_0,*te*,*ij*_ is the tentative estimates of the range information based on *i*th up ramp beat frequency and *j*th down ramp beat frequency and *v*_*te*,*ij*_ is the tentative estimates of the velocity information based on *i*th up ramp beat frequency and *j*th down ramp beat frequency. After inserting *R*_0,*te*,*ij*_ and *v*_*te*,*ij*_ into Eq. (9), the estimated check beat frequency based on *i*th up ramp beat frequency and *j*th down ramp beat frequency can be formulated as

12 $$\hat{f}_{bc,ij} = \frac{{2BR_{0,te,ij} }}{{cT_{2} }} + \frac{{2f_{c} v_{te,ij} }}{c}.$$

Using the above, we can perform the following procedure to determine if the target is a real target or a ghost target:

1. If the difference between $\hat{f}_{bc,ij}$ and *f*_*bc*,*k*_ is smaller than a threshold *ε*_*f*_, the tentative estimates of range information *R*_0,*te*,*ij*_ and velocity information *v*_*te*,*ij*_ are the real target information. The threshold *ε*_*f*_ needs to be selected carefully between the tolerance of system errors, and the rejection of ghost targets. In practice, trails of taking real measurements and evaluating the corresponding detection performance must be done to determine the threshold.
2. If the difference between $\hat{f}_{bc,ij}$ and *f*_*bc*,*k*_ is larger than *ε*_*f*_, the tentative estimates of range information *R*_0,*te*,*ij*_ and velocity information *v*_*te*,*ij*_ are the ghost target information.

Through the above procedures, the range and velocity information of real targets can be obtained, and most of the range and velocity information of ghost targets can effectively be reduced. However, if the number of targets is large, the pairing process is complex and unreliable. In the next section, the proposed detection architecture and algorithms will be introduced to solve this problem.

In the automotive radar systems, the range resolution Δ*R* represents the minimum discernible range of two targets with the same velocity, and the velocity resolution Δ*v* represents the minimum discernible velocity of two targets with the same range. Consider the system specifications as given in Table 1 for a practical automotive radar application. The corresponding parameters of the three-segment waveform can be obtained as follows. According to (Rohling and Meinecke 2001), the bandwidth *B* is related to the given range resolution Δ*R* and can be formulated as

13 $$B = \frac{c}{2\varDelta R}.$$

**Table 1:** The system specifications

Parameter	Long-range automotive radar	Short-range automotive radar
Maximum range *R*_Max_	200 (m)	50 (m)
Maximum velocity *v*_Max_	200 (km/h)	150 (km/h)
Range resolution Δ*R*	1 (m)	0.1 (m)
Velocity resolution Δ*v*	1 (km/h)	1 (km/h)

Similarly, the observation time *T* is related to the velocity resolution Δ*v* and can be expressed as

14 $$T = \frac{c}{{2f_{c} \varDelta v}}.$$

Finally, according to the Nyquist sampling theorem and the Eqs. (13) and (14), the sampling frequency is

15 $$f_{s} \ge \frac{{2BR_{\hbox{max} } }}{cT} + \frac{{2f_{c} v_{\hbox{max} } }}{c}.$$

The parameters of the three-segment waveform are summarized in Table 2. The measurement times in (Rohling and Moller 2008; Rohling and Meinecke 2001; Miyahara 2004; Bi and Du 2010) are 10, 10, 100 and 5 ms, respectively. The measurement time of classical FMCW waveforms is about 50 ms (Rohling and Meinecke 2001). Our measurement time is only 24 ms (2 × T_1_ + T_2_.) To adopt FMCW with another modulation waveform (Rohling and Moller 2008; Rohling and Meinecke 2001; Bi and Du 2010) or do more sweeps in one measurement cycle (Miyahara 2004) will potentially increase burdens of RF waveform generators. Our approach is to keep the generation of FMCW waveforms simple. The architecture and algorithms for multi-target detections will be described in the next section.

**Table 2:** The corresponding parameters of the three-segment waveform

Parameter	Long-range automotive radar	Short-range automotive radar
Sampling rate *f*_*s*_	150 (kHz)	88 (kHz)
Bandwidth *B*	150 (MHz)	1500 (MHz)
Ramp duration *T*_1_, *T*_2_	7, 10 (ms)	7, 10 (ms)

## Multi-target detection architecture and algorithms

**Fig. 4:**
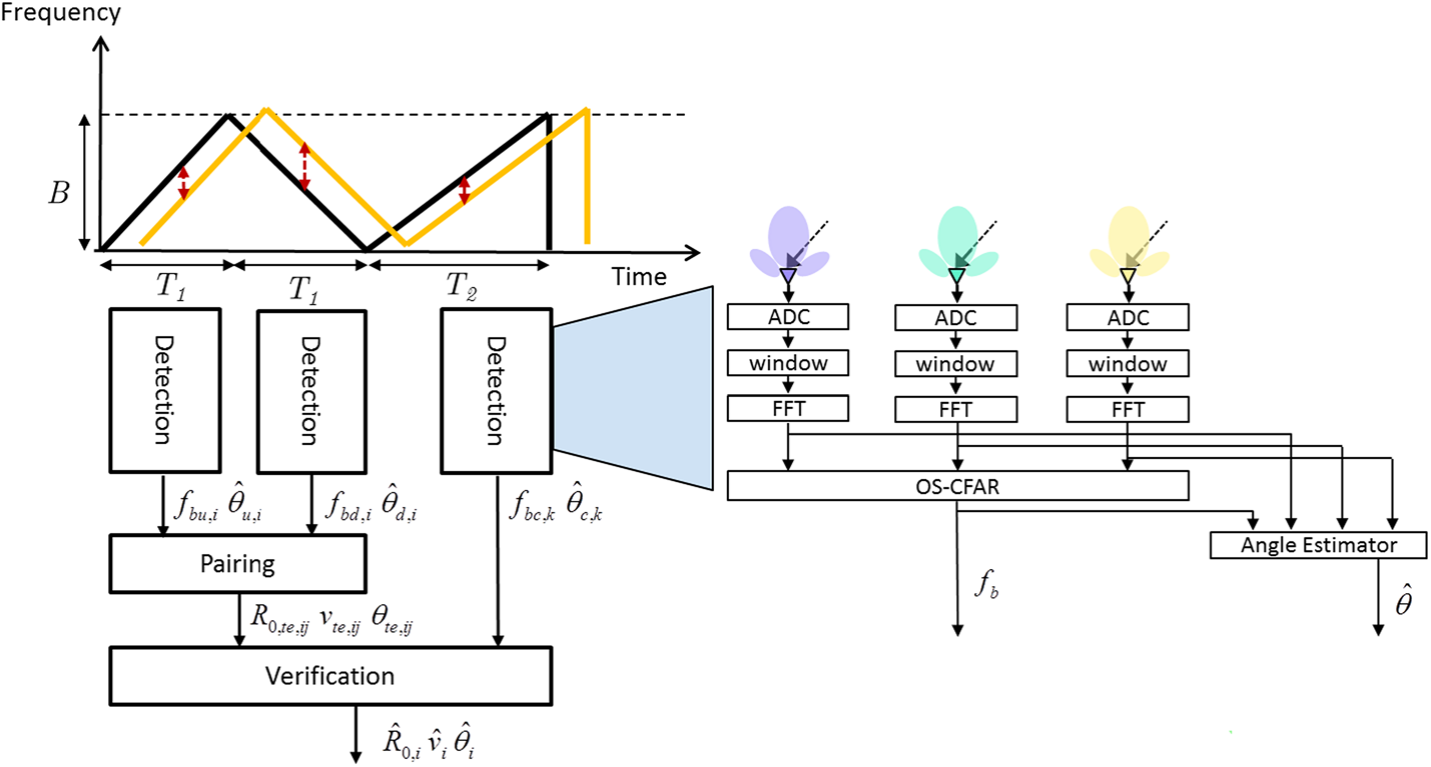
The proposed multi-target detection architecture

To deal with the complexity of pairing process and the unreliability of the estimated range, velocity, and angle information, new detection architecture and algorithms are discussed in this section. The proposed detection architecture shown in Fig. 4 is made up of the detection process, the pairing process, and the verification process, as follows:

1. In the detection process, the received signal is multiplied by the window function and transformed into frequency domain by utilizing the FFT operation. After the OS-CFAR (Rohling 1983) process, the beat frequencies can be obtained. Then, the angle information can also be estimated based on the phase difference of the signals received by receiving antennas at the beat frequency (Huang et al. 2013).
2. In the pairing process, only the beat frequencies of the up ramp *f*_*bu*,*i*_ and down ramp *f*_*bd*,*j*_ with similar angle information $\hat{\theta }_{u,i}$ and $\hat{\theta }_{d,j}$ are paired to calculate the corresponding tentative estimates of the range *R*_0,*te*,*ij*_, velocity *v*_*te*,*ij*_ and angle *θ*_*te*,*ij*_ information.
3. In the verification process, in addition to the difference between the estimated check beat frequency $\hat{f}_{bc,ij}$ and the real check beat frequency *f*_*bc*_, the estimated and real angle information *θ*_*te*,*ij*_ and *θ*_*c*_ is also considered.

Through the above-mentioned flow, the proposed detection architecture can effectively reduce pairing time and enhance the system reliability.

**Fig. 5:**
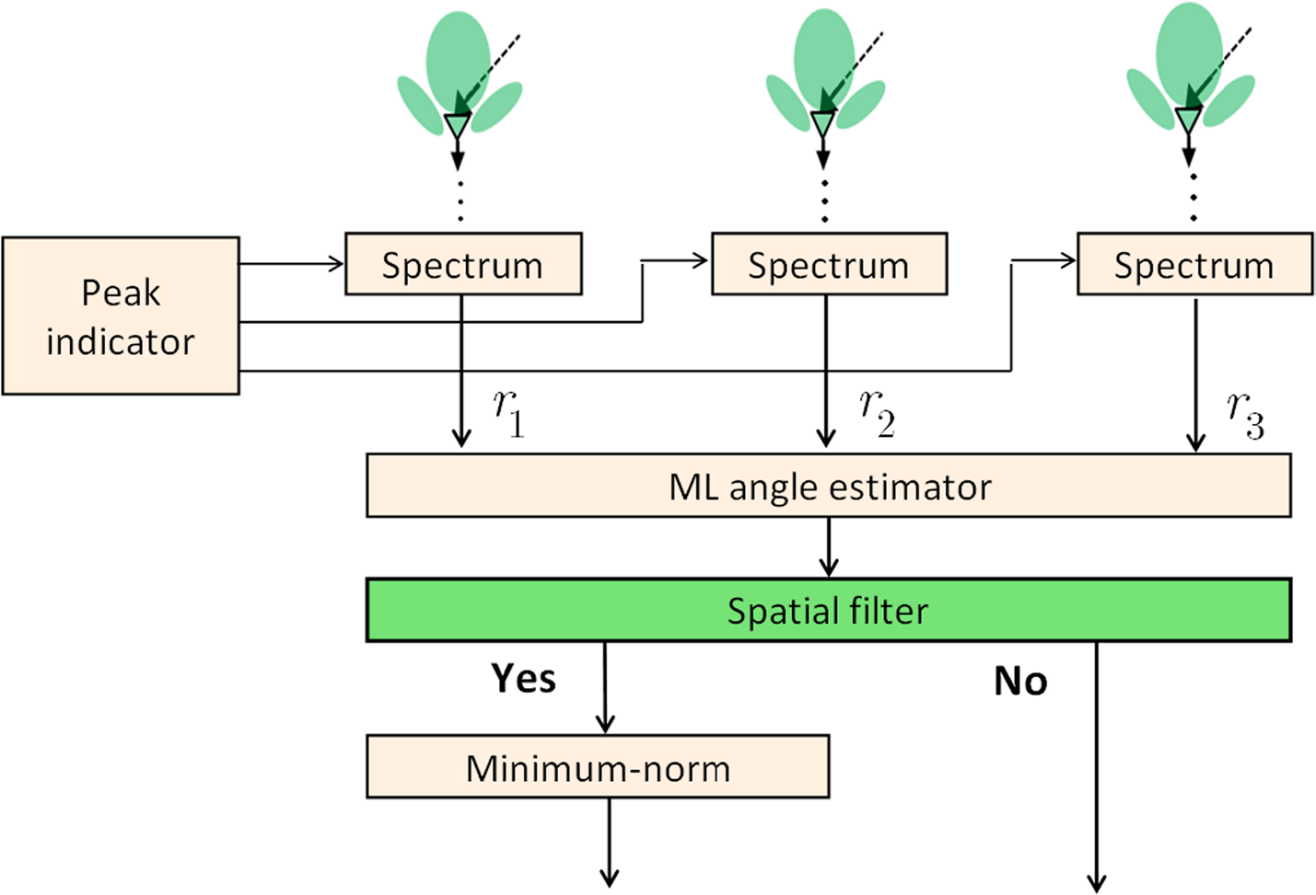
The proposed multi-target angle estimation process

For angle estimation, (Huang et al. 2013) provides an accurate and low-complexity solution. When the estimated angle information overlaps between beat frequencies of different targets, the angle information might be unreliable. To deal with such overlapping problems, the method which incorporates with the minimum-norm method and the spatial filter algorithm is proposed. The whole angle estimate architecture and process is shown in Fig. 5. In (Häcker and Yang 2010), the eigen-based angle estimator (e.g. root-multiple signal classification (root-MUSIC), minimum-norm, and estimation of signal parameters via rotational invariance technique (ESPRIT)) can separate targets with a similar beat frequency in a single snapshot. When compared to the root-MUSIC, the minimum-norm method requires lower computational complexity and has comparable performance when the number of antennas is small (Rao and Hari 1989). Thus, the minimum-norm method is adopted in our system. However, if each estimated angle is processed through the minimum-norm method, the computational effort will be extremely high, because a lot of wrong angle information is generated and used in the pairing and verification process. Therefore, the spatial filter design algorithm is proposed to determine the overlapping initially. This will lead to lower computation loading and better accuracy with the minimum-norm method applied. Considering three receive antennas with an equal spacing *d*_*R*_, the steering vector of the received signals can be expressed as

16 $${\mathbf{a}}(\phi ) = \left[ {\begin{array}{*{20}c} 1 & {e^{{j\tfrac{{2{{\pi }}}}{\lambda }d_{R} \sin (\phi )}} } & {e^{{j\tfrac{{2{{\pi }}}}{\lambda }2d_{R} \sin (\phi )}} } \\ \end{array} } \right]^{\text{T}} ,$$

where $\phi$ is the incoming angle, *λ* is denoted as the wave length, and $\varvec{[ } \cdot \varvec{ ]}^{\text{T}}$ denotes the vector transpose operator. After passing **a**($\phi$) through the spatial filter, the output value *y*, which is the inner product of **a**($\phi$) and a weight vector ${\mathbf{w}}_{d} = \left[ {w_{1} \, w_{2} \, w_{3} } \right]^{\text{T}}$ of the spatial filter, can be expressed as a quadratic form

17 $$ \begin{aligned} y &= {\mathbf{w}}^{\text{T}} {\mathbf{a}}\left( \phi \right) \hfill \\ & = \sum\limits_{i = 0}^{2} {w_{i} z^{i} } \hfill \\ & = c(z - z_{1} )(z - z_{2} ), \hfill \\ \end{aligned} $$

where $z = e^{{j\tfrac{{2{{\pi }}}}{\lambda }d_{R} \sin (\phi )}}$. Let *c* = 1, $z_{1} = e^{{j\tfrac{{2{{\pi }}}}{\lambda }d_{R} \sin (\phi )}}$, and $z_{2} = e^{{j\tfrac{{2{{\pi }}}}{\lambda }d_{R} \sin (\phi + \delta )}}$, the weight vector of the spatial filter can be expressed as

18 $${\mathbf{w}}_{d} = \left[ {\begin{array}{*{20}c} 1 & { - \left( {e^{{j\tfrac{{2{{\pi }}}}{\lambda }d_{R} \sin (\phi )}} + e^{{j\tfrac{{2{{\pi }}}}{\lambda }d_{R} \sin (\phi + \delta )}} } \right)} & {e^{{j\tfrac{{2{{\pi }}}}{\lambda }d_{R} \left[ {\sin (\phi ) + \sin (\phi + \delta )} \right]}} } \\ \end{array} } \right]^{\text{T}} .$$

Hence, only the estimated angles whose output values of the spatial filter are larger than a threshold *ε*_*S*_ need to be processed through the minimum-norm method, as shown in Fig. 5. Note that the threshold *ε*_*S*_ needs to be selected carefully among the tolerance of system errors, the accuracy of the angle estimation, and the computational loading. The proposed multi-target angle estimation process can solve the angle ambiguity and overlapping problems with low complexity and high accuracy.

## Simulation and experiment results

In this section, the performance of the proposed baseband signal processing architecture and algorithms is evaluated by computer simulations and field measurements.

### Simulation results

**Fig. 6:**
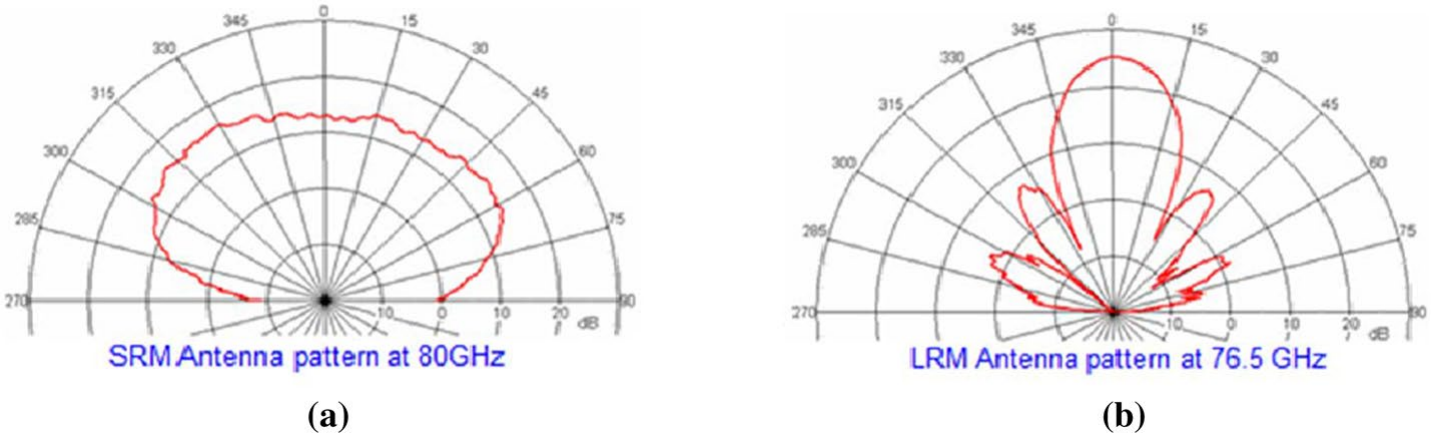
**a** The antenna pattern of the short-range (SR) automotive radar. **b** The antenna pattern of the long-range (LR) automotive radar

Computer simulations are conducted with five steps as follows:

1. The random traffic pattern is generated by the random target generating function. This emulates the multi-target detection scenario in a five-lane highway.
2. Both of the SR and LR automotive radars are simulated with the antenna patterns as shown in Fig. 6. Note that the FOVs (field of view) of the SR and LR automotive radars are −45° to +45° and −8° to +8°, respectively. The simulation parameters are listed in Table 3.

**Table 3:** Parameters for computer simulations

Parameter	Value
Long-range automotive radar	
Operating frequency	77 GHz
Transmit power	23 dBm
Noise power	−119.5 dBm
Noise figure	12.8 dB
Number of receiving antennas	3
Antenna spacing	1.5*λ*
Field of view	−8° to +8°
Short-range automotive radar	
Operating frequency	77 GHz
Transmit power	12 dBm
Noise power	−119.5 dBm
Noise figure	12.8 dB
Number of receiving antennas	3
Antenna spacing	0.6*λ*
Field of view	−45° to +45°
3. The radar cross section (RCS) of a car as shown in Fig. 3 of (Hasch et al. 2012) is adopted.
4. The detection results for the short-range (SR) and long-range (LR) automotive radars are simulated through using the random traffic pattern (shown in Figs. 7, 8). The velocities, ranges and angles are also randomly assigned to each target.

**Fig. 7:**
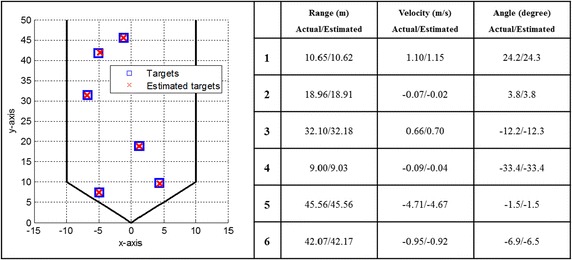
The detection result for the short-range automotive radar

**Fig. 8:**
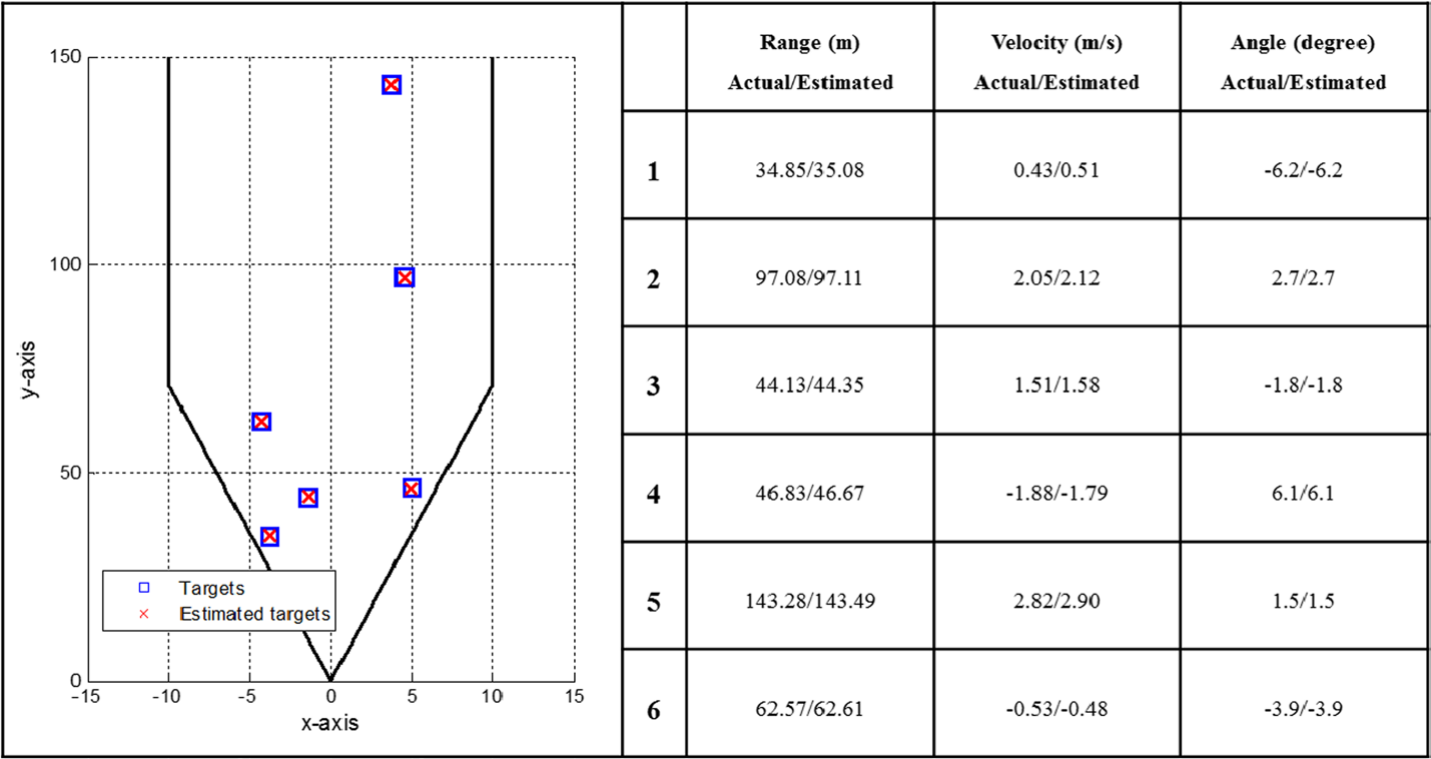
The detection result for the long-range automotive radar
5. The detection probability and the root mean-squared errors (RMSE) simulated for the SR automotive radar with different number of targets are shown in Figs. 9, 10, respectively.

**Fig. 9:**
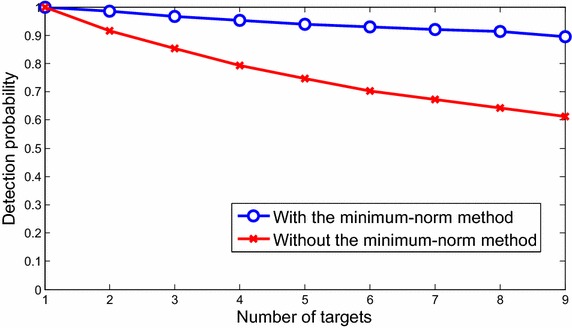
The detection probability for the short-range automotive radar

**Fig. 10:**
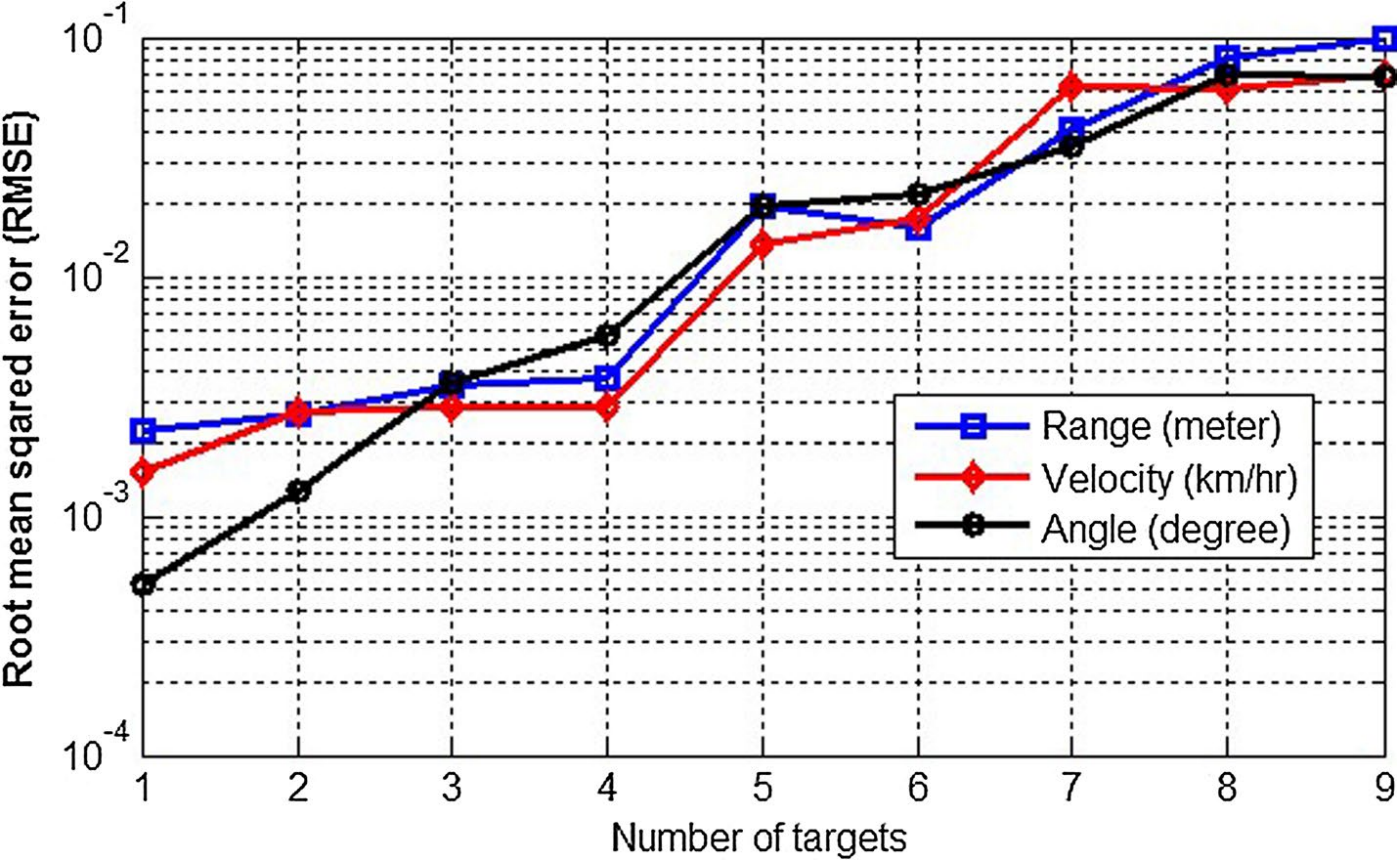
The root mean squared error (RMSE) for the short-range automotive radar

The random traffic patterns generated with six targets are simulated in the SR and LR automotive radars, respectively. First, the detection result for the SR automotive radar is shown in Fig. 7. It is clearly observed that the proposed multi-target detection architecture and algorithms can precisely detect multiple targets in the SR automotive radar. Similar detection result for the LR automotive radar is shown in Fig. 8. These detection results demonstrate that the proposed multi-target detection architecture and algorithms can accurately detect multiple targets in a single snapshot for the SR or LR automotive radar.

For more statistical analyses, the detection probability for the SR automotive radar with different number of targets is shown in Fig. 9, where the detection probability is defined as the ratio of the number of detected real targets to the number of total real targets. The result shows that the detection probability curve will significantly increase with the minimum-norm method, because the minimum-norm method can separate targets with similar beat frequencies in a single snapshot. Furthermore, RMSE of estimated ranges, velocities and angles are shown in Fig. 10, with respect to random process, environment, and sounding signal parameters. The RMSEs increase with the larger number of targets. This means the more targets, the more ambiguity. Therefore, as shown in Figs. 9 and 10, the detection probability will decrease and the RMSE will increase as more targets appear.

**Fig. 11:**
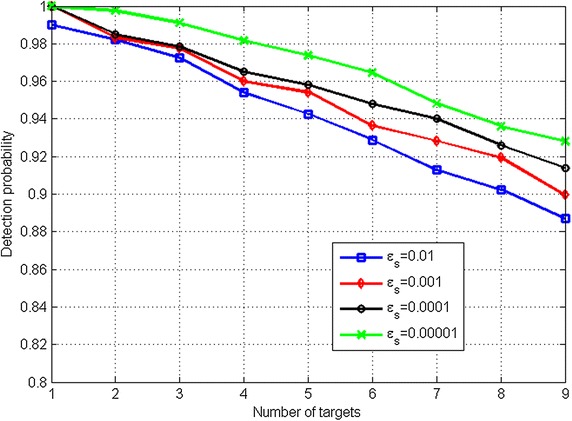
The detection probability vs. different *ε*_*s*_ values for the short-range automotive radar

Furthermore, as the threshold (*ε*_*S*_) discussion in “Multi-target detection architecture and algorithms”, the *ε*_*S*_ value can be selected to obtain a higher detection result from the random traffic pattern simulations. The smaller *ε*_*S*_ value (less filtering) will lead to a higher detection probability (Fig. 11), but a higher computation loading. In this paper, the *ε*_*S*_ value is set as 0.0001 in both simulations and field measurements to obtain the balance between the estimation accuracy and computation loading.

### Field measurements

Next, field measurements are conducted to assess the efficacy of the proposed method. Different from the 77-GHz setting used in the computer simulations, an existing and verified 24-GHz automotive radar module (Ho and Chung 2005) is adopted for measurements. The same system parameters as shown in Table 2 are used. With the 24-GHz carrier frequency and using the parameters of observation time *T* = 7 ms, bandwidth *B* = 150 MHz, and sampling rate *fs* = 150 kHz, the range resolution maintains Δ*R* = 1 m but the velocity resolution changes to Δ*v* = 3.2 km/h, according to Eqs. (13)–(15).

**Fig. 12:**
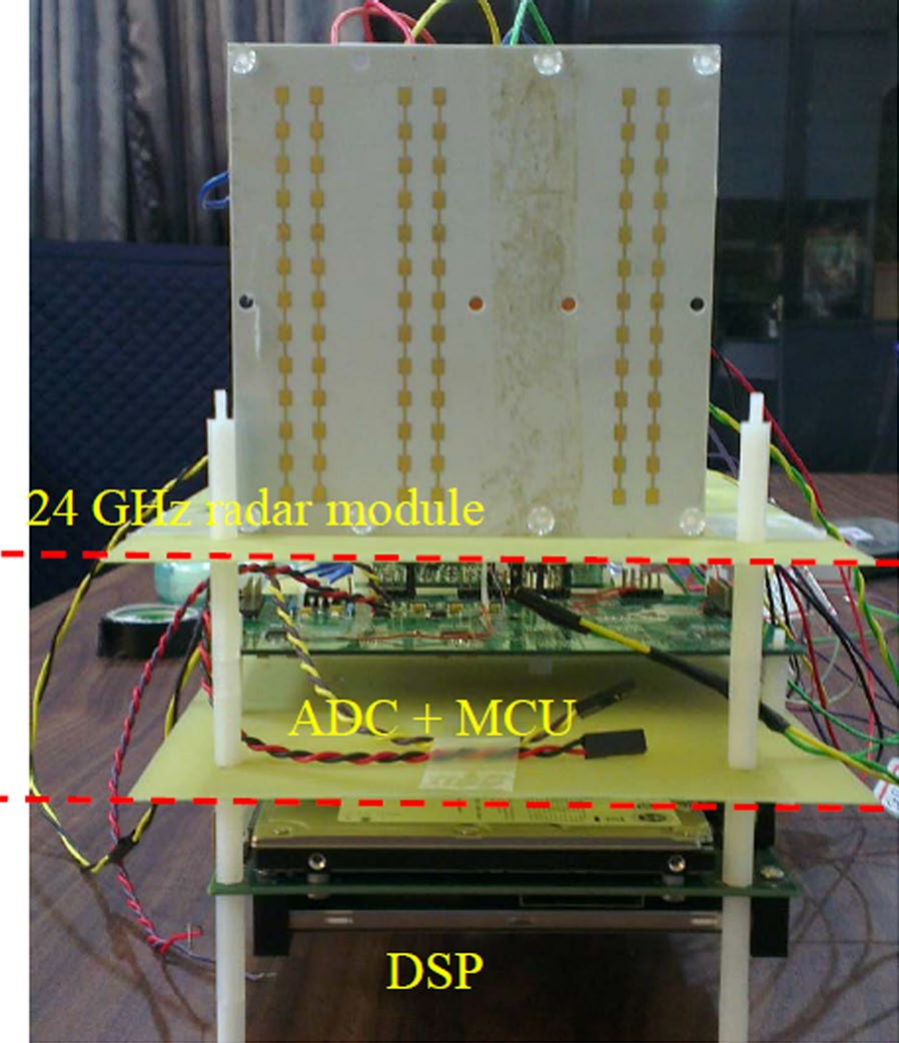
A 24-GHz radar module with an ADC module and a DSP evaluation board for baseband signal processing. *MCU* micro-controller unit

**Fig. 13:**
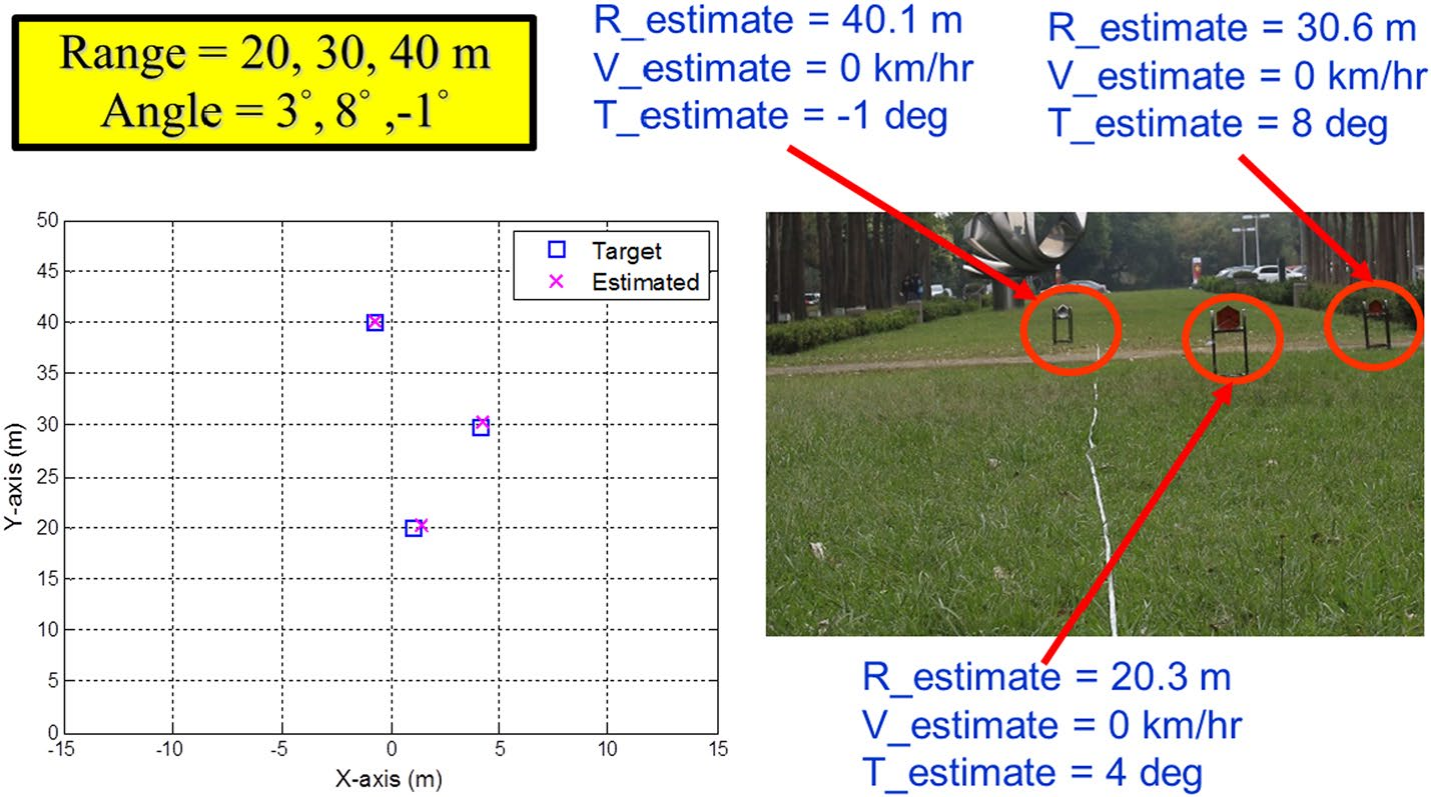
The field measurement result for the long-range automotive radar

Figure 12 shows the 24-GHz radar module with an ADC (analog-to-digital converter) module and a DSP (digital signal processor) evaluation board. The proposed baseband signal processing architecture and algorithms are implemented in the DSP board. Note that due to the limitation of the system bandwidth and the physical memory size for the 24-GHz automotive radar module, only the LR radar algorithm was executed for the field measurements. In addition, the angle resolution was limited to 1°. This is due to the fact that the angle estimator is implemented on hardware using look-up tables which will lead to an error resolution of 1°. Figure 13 shows the field measurement results for the LR automotive radar. Three targets located at National Chiao Tung University (Taiwan) were measured with

1. *R* = 20.0 m, *v* = 0 km/h, and *θ* = 3°;
2. *R* = 30.0 m, *v* = 0 km/h, and *θ* = 8°;
3. *R* = 40.0 m, *v* = 0 km/h, and *θ* = −1°.

The measurement results were respectively

1. *R* = 20.3 m, *v* = 0 km/h, and *θ* = 4°;
2. *R* = 30.6 m, *v* = 0 km/h, and *θ* = 8°;
3. *R* = 40.1 m, *v* = 0 km/h, and *θ* = −1°.

According to the field measurement results, the range deviations were less than 1 m and the angle deviations were less than 1°. The range estimation meets the system specifications in Table 1. The velocity deviations were less than Δ*v* = 3.2 km/h, which also meet the aforementioned 24-GHz specification. Furthermore, the date refresh rate meets the target as 25 ms (2 × T_1_ + T_2_ + a 1-ms guard period) in practice. The computer simulation and field measurement results demonstrate that the proposed baseband signal processing architecture and algorithms for the automotive radar system can in fact perform well and meet the system specifications.

## Conclusions

This paper proposes the baseband signal processing architecture and algorithms in the automotive radar systems. To satisfy the short measurement time constraint without increasing the RF front-end loading, the three-segment waveform is adopted, which incorporates the check ramp to verify the paring information and increase the system reliability. By given the system specification, the corresponding parameters of three-segment waveform are also discussed. To overcome the ghost target and overlapping problem, the detection architecture and algorithms are proposed. The proposed multi-target detection architecture, which is composed of the detection, the pairing, and the verification processes, can effectively reduce pairing time and enhance the system reliability. Moreover, the proposed multi-target angle estimation process is able to resolve the overlapping problem while having low pairing time and high reliability. Finally, simulation and field measurement results demonstrate that the proposed detection architecture and algorithms can reliably detect multiple targets in the automotive radar systems. The proposed architecture and algorithms balance the performance and the complexity, and are extremely suitable to be implemented in a practical automotive radar system.
